# Mental Illnesses and Quality of Sleep Among Nurses Working at a Tertiary Hospital in Riyadh During the COVID-19 Pandemic

**DOI:** 10.7759/cureus.47394

**Published:** 2023-10-20

**Authors:** Ahmed F El Fouhil, Yazeed F Aldakheel, Ibrahim S Alnamlah, Hesham A Alsqabi, Abdulmuhsen H Alfaifi

**Affiliations:** 1 Department of Anatomy, King Saud University Medical City, Riyadh, SAU; 2 College of Medicine, King Saud University Medical City, Riyadh, SAU

**Keywords:** cross-sectional study, nurses, covid-19, quality of sleep, mental health

## Abstract

Objectives

This study primarily aimed to estimate the prevalence of mental illnesses (depression, anxiety, and stress) along with the poor quality of sleep, with a secondary focus on determining whether there was an association between mental health and quality of sleep among nurses working at King Khalid University Hospital, Riyadh, Saudi Arabia, during the COVID-19 pandemic.

Methods

A cross-sectional study was conducted from June to December 2021 during the COVID-19 pandemic on 309 nurses from different departments at King Khalid University Hospital. Depression, anxiety, and stress were measured by the Depression, Anxiety, and Stress Scale - 21 Items (DASS-21), and sleep quality was measured by the Pittsburg Sleep Quality Index (PSQI) via an online survey.

Results

The prevalence of depression, anxiety, and stress was 30.4%, 43.7%, and 16.5%, respectively, while 61.5% showed poor sleep quality. The association between poor quality of sleep and the other outcome variables of mental health (depression, anxiety, and stress) was highly significant (p<0.0001).

Conclusion

Nurses have reported an increased prevalence of depression, anxiety, stress, and poor quality of sleep during the COVID-19 pandemic, making them particularly vulnerable to mental illnesses and sleep difficulties in the event of future pandemics. There is a clear link between mental health issues and poor quality of sleep, necessitating the provision of psychological support for nurses. Enhancing sleep quality is recommended as a way to decrease the prevalence of mental health concerns.

## Introduction

The COVID-19 pandemic has impacted life worldwide. According to the WHO, around 6.6 million deaths were globally recorded, and that number is rising [1]. The pandemic has also placed immense pressure on healthcare workers, increasing their workload, mental strain, and social isolation, making them more susceptible to its negative effects [2]. Owing to their prolonged contact with infected patients together with their fear of infection, nurses are more vulnerable to stress and depression. These factors, along with anxiety and sleep disorders, have a negative impact on the psychophysical health of nurses, which may affect professional performance and patient safety [3,4]. As nurses play an important role in the healthcare team, their mental health and quality of sleep are crucial while facing this crisis so any negative effect on them would reflect on their professional performance.

In Riyadh, Saudi Arabia, a cross-sectional study was conducted on nurses in January 2021, and it showed a prevalence of depression symptoms in 29.2% of participants, of anxiety symptoms in 57%, and of stress symptoms in 12% [5]. In another Saudi study, performed in central, eastern, and western regions, with a sample of 1130 healthcare workers, of which 262 were nurses, showed that 19.8% of nurses suffered from moderate to severe depression, and 14.9% suffered from moderate to severe anxiety [6]. An online study, in Egypt and Saudi Arabia, was conducted on 426 healthcare workers, and of them, 103 were nurses. The study reported mild to very severe depression in 65% of nurses, mild to very severe anxiety in 59.3%, and mild to very severe stress in 53.4% [7]. On the other side of the globe in California, USA, a cross-sectional study including 320 nurses, performed during the COVID-19 pandemic, demonstrated that 26% of the participants suffered from moderate to severe depression, 43% suffered from moderate to severe anxiety, and 80.1% suffered from moderate to severe stress [8].

In April 2020, in Guilan province, Iran, a study conducted on 441 nurses, the majority of whom were in contact with suspected or confirmed cases of COVID-19, showed depression and anxiety in 37.4% and 38.8% of the participants, respectively [9]. A Chinese study reported that 39.91% of the respondent nurses had stress [10]. A cross-sectional study was conducted on 238 nurses at Al-Madinah University, in Almadinah Almunawwarah, Saudi Arabia, during the first year of the COVID-19 crisis. Using the Pittsburg Sleep Quality Index (PSQI), the study demonstrated that 65.6% of the participants had poor sleep quality [11]. Another Chinese research conducted on 100 frontline nurses, in February 2020, revealed that 60% of the nurses had poor sleep quality, 46% had depression symptoms, and 40% had anxiety symptoms [12].

A descriptive study in Turkey investigated the quality of sleep among 105 nurses working in a state hospital, using the PSQI, and revealed that 70.9% of nurses experienced poor sleep quality [13]. A Chinese cross-sectional study examined the relationships between mental health problems and sleep difficulty among 323 nurses. It has been shown that nurses with higher levels of mental health problems suffer from more sleep difficulty [14]. Similarly, there was a high prevalence of poor sleep quality and, hence, impairment of psychological health among nurses, in a study performed in Turkey [15].

The present study primarily aimed at estimating the prevalence of mental illnesses (depression, anxiety, and stress) along with poor quality of sleep with a secondary focus on determining whether there was an association between mental illnesses and quality of sleep among nurses working in Saudi Arabia during the COVID-19 pandemic. To the best of our knowledge, studies conducted in Saudi Arabia investigating mental health and quality of sleep among nurses are scarce, especially in Riyadh. Being the capital, it has the highest population and highest number of infected individuals in Saudi Arabia. King Khalid University Hospital (KKUH), one of the leading governmental hospitals in Riyadh, was the preferred choice for performing such a study.

The results of this study hopefully provided sufficient information about mental health and quality of sleep among nurses, two years following the start of the COVID-19 pandemic, giving us a greater understanding of how to deal with future ones while minimizing their negative effects. Moreover, raising awareness of mental illnesses and poor quality of sleep will be beneficial for nurses and for future researchers interested in this field.

## Materials and methods

The study was approved on the third of August 2021 by the Institutional Review Board, College of Medicine, King Saud University (E-21-6169). All the procedures followed the ethical standards of which the consent was perceived. All participants' information was kept confidential.

The present study is a descriptive cross-sectional study that used an online survey. This study was conducted over six months, from June to December 2021, during the COVID-19 pandemic. The subjects were nurses working at the KKUH, Riyadh, Saudi Arabia. Of them, 309 were included from different departments, using a snowball sampling technique. The inclusion criteria were nurses working at KKUH. Exclusion criteria were nurses on leave at KKUH in 2021 during data collection.

The online questionnaire was constructed on Google Forms and sent by e-mail to the head nurses of different departments who were instructed to share it with nurses to fill it out.

With an assumed response rate of 80%, a 95% confidence interval, and a 5% error margin, the minimum required sample size is (n = 257). The data of 309 nurses were collected between September 21, 2021, and October 13, 2021. The survey was comprised of four major parts; the first and second were the exposure variables of the participants. The first part was the sociodemographic information including gender, age, nationality, marital status, work experience, working hours per day, clinical department, and whether they were living alone or not.

The second part was exposure to COVID-19. It was measured by asking nurses about the frequency of contact with COVID-19 patients (four-point response scale from never to very often) [16] and whether they were infected with COVID-19 or not. The third and fourth parts were the outcome variables which were the symptoms of depression, anxiety, and stress that were assessed using the validated Depression, Anxiety, and Stress Scale - 21 Items (DASS-21) [17], as well as the quality of sleep, which was assessed using the PSQI [18].

DASS-21 is a collection of three self-report scales that assess depression, anxiety, and stress. Each of the three scales has seven items grouped into subscales that have comparable content. The depression scale assesses dysphoria, hopelessness, devaluation of life, self-deprecation, lack of interest/involvement, anhedonia, and inertia. The anxiety scale assesses autonomic arousal, skeletal muscle effects, situational anxiety, and subjective experience of anxious affect. The stress scale is sensitive to levels of chronic nonspecific arousal. It assesses difficulty relaxing, nervous arousal, being easily upset/agitated, irritable/over-reactive, and impatient. The participants are asked to rate how much the statements applied to them over the past week, on a four-point scale from 0 (did not apply to me at all) to 3 (applied to me most of the time). Scores are calculated by summing the values and multiplying them by two for the relevant seven items and then categorized by cutoff point into normal, moderate, and severe [17].

The Pittsburgh Sleep Quality Index (PSQI) is a 19-item self-reported questionnaire that evaluates sleep quality and disturbances over the last month. Subjective sleep quality, sleep latency, sleep duration, habitual sleep efficiency, sleep disturbances, usage of sleeping medication, and daytime dysfunction are among the 19 individual items that create seven "component" scores. The total of these seven components' scores equals one global score (0-21), where more than five is considered poor quality of sleep [18].

Data were analyzed using Statistical Product and Service Solutions (SPSS) (version 24.0; IBM SPSS Statistics for Windows, Armonk, NY). Descriptive statistics (frequencies, percentages, mean, and standard deviation) were used to describe the categorical and quantitative variables. Univariate analysis was conducted using the student's t-test for independent samples. Moreover, Pearson’s chi-square test was used to assess the association between categorical study and outcome variables. Odds ratios were calculated to measure association. A p-value of ≤0.05 and 95% confidence intervals will be used to report the statistical significance and precision of the results.

## Results

Out of 309 nurses, 275 (89%) were females and 34 (11%) were males. The frequency of contact with COVID-19 patients was never for 27 (8.7%), rarely for 160 (51.8%), often for 81 (26.2%) and very often for 41 (13.3%). Of them, 66 (21.4%) have been infected with COVID-19. The distribution of other sociodemographic characteristics is shown in Table 1.

**Table 1:** Sociodemographic data of nurses working at KKUH (n=309).

Variable	Mean (sd)
Working hours	11.16 (1.476)
Age	N (%)
Less than 36	158 (51.1)
36 to 45	98 (31.7)
More than 45	53(17.2)
Gender	N (%)
Female	275 (89)
Male	34 (11%)
Nationality	N (%)
Filipino	191 (61.8)
Indian	95 (30.7)
Others	23 (7.4)
Marital status	N (%)
Married	208 (67.3)
Unmarried	101 (32.7)
Work experience	N (%)
Less than 10 years	134 (43.4)
10 to 20 years	143 (46.3)
More than 20 years	32 (10.4)
Clinical department	N (%)
Medicine	87 (28.2)
Ambulatory care	52 (16.8)
ICU	51 (16.5)
Women’s health and pediatric	40 (12.9)
Surgery	36 (11.7)
Others	43 (13.9)
Living states	N (%)
Alone	99 (32)
Not alone	210(67.9)
Frequency of contact with COVID-19 patients	N (%)
Never	27 (8.7)
Rarely	160 (51.8)
Often	81 (26.2)
Very often	41 (13.3)
COVID-19 infection	N (%)
Yes	66 (21.4)
No	243 (78.6)

Ninety-four (30.4%) participants had symptoms of depression; of them, 54 (17.5%) had moderate depression, and 40 (12.9%) had severe depression, while 215 (69.6%) had no symptoms of depression, and they were considered normal. Symptoms of anxiety were evident in 135 (43.7%); 64 (20.7%) of them had moderate anxiety, 71 (23%) had severe anxiety, and 174 (56.3%) were normal. Symptoms of stress were evident in 51 (16.5%) nurses; 19(6.1%) of them had moderate stress, and 32 (10.4%) had severe stress, while 258 (83.5%) had no symptoms of stress. One hundred ninety (61.5%) participants had poor quality of sleep, while 149 (38.5) were normal (Table 2).

**Table 2:** Prevalence of depression, anxiety, stress, and poor quality of sleep.

Variable	N(%)
Depression (0-42)	
Normal (0-13)	215 (69.6)
Moderate (14-20)	54 (17.5)
Severe (21-42)	40 (12.9)
Anxiety (0-42)	
Normal (0-9)	174 (56.3)
Moderate (10-14)	64 (20.7)
Severe (15-42)	71 (23)
Stress (0-42)	
Normal (0-18)	258 (83.5)
Moderate (19-25)	19 (6.1)
Severe (26-42)	32 (10.4)
Poor quality of sleep	190 (61.5)

The association between the poor quality of sleep and the other outcome variables of mental health (depression, anxiety, and stress) was highly significant (p<0.0001); depression (Χ2=29.021, OR=4.935, 95% CI of OR=2.672, 9.113), anxiety (Χ2=22.199, OR=3.219, 95% CI of OR=1.961, 5.284), and stress (Χ2=13.438, OR=4.059, 95% CI of OR=1.835, 8.978) (Table 3).

**Table 3:** Relation between depression, anxiety, stress, and quality of sleep.

Variables	Quality of sleep N(%)		Χ^2^ value	p-value	OR	95% CI of OR
	Poor	Good				
Depression			29.021	<0.0001	4.935	2.672, 9.113
With symptoms	79 (840	15 (16)				
Normal	111 (51.6)	104 (48.4)				
Anxiety			22.199	<0.0001	3.219	1.961, 5.284
With symptoms	103 (76.3)	32 (23.7)				
Normal	87 (50)	87 (50)				
Stress			13.438	<0.0001	4.059	1.835, 8.978
With symptoms	43 (84,3)	8 (15.7)				
Normal	147 (57)	111 (43)				

Symptoms of depression were more likely to occur in nurses younger than 36 years of age (OR=1.853, 95% CI=1.130, 3.039), unmarried nurses (OR=2.608, 95% CI=1.571, 4.327), and nurses that have less than 10 years of experience (OR=1.886, 95% CI=1.156, 3.079). There was no statistical significance between the depression and other study variables, as shown in Table 4.

**Table 4:** Relation between depression, anxiety, and study variables.

Variables	P value	Depression OR	95% CI of OR		Anxiety	
				P value	OR	95% CI of OR
Age (years)	0.014	1.853	1.13, 3.039	<0.0001	2.486	1.565, 3.949
<36						
>36						
Gender	0.066	0.511	0.247, 1.055	0.024	0.438	0.211, 0.911
Female						
Male						
Nationality	0.036			0.071		
Filipino		1 (Reference)			1 (Reference)	
Indian		1.890	1.061, 3.367		1.639	0.985, 2.728
Others		0.655	0.272, 1.575		0.671	0.281, 1.605
Marital status	<0.0001	0.384	0.231, 0.636	0.004	0.492	0.304, 0.797
Married						
Not married						
Work experience (years)	0.011	1.886	1.156, 3.079	0.016	1.753	1.111, 2.767
<10						
>10						
Clinical department	0.346			0.019		
Medicine		1 (Reference)			1 (Reference)	
Ambulatory care		1.143	0.522, 2.504		1.321	0.642, 2.719
ICU		0.642	0.307, 1.341		0.570	0.284, 1.146
Women’s health and pediatric		1.312	0.545, 3.159		1.332	0.605, 2.934
Surgery		0.533	0.237, 1.202		0.363	0.162, 0.811
Others		0.789	0.357, 1.743		0.672	0.322, 1.404
Live status	0.303	1.308	0.784, 2.181	0.854	1.046	0.647, 1.693
Alone						
Not alone						
Frequency of contact with COVID-19 patients	0.977	0.993	0.605, 1.630	0.690	1.098	0.693, 1.738
Often/Very often Never/Rarely						
COVID-19 Infection	0.981	0.993	0.549, 1.794	0.545	1.184	0.686, 2.044
Infected						
Not infected						
Working hours	t-value	P-value	95% CI of deference mean	t-value	P-value	95% CI of deference mean
	0.677	0.499	-0.236, 0.483	2.077	0.039	0.018, 0.681

Anxiety symptoms were more likely to manifest in nurses who were younger than 36 years of age (OR=2.486, 95% CI=1.565, 3.949), unmarried nurses (OR=2.032, 95% CI=1.255, 3.291), and nurses with less than 10 years of experience (OR=1.753, 95% CI=1.111, 2.767). There was no statistical significance between anxiety and the other study variables, as shown in Table 4.

Symptoms of stress were more likely to be exhibited by nurses younger than 36 years of age (OR=2.675, 95% CI= 1.397, 5.125) and unmarried nurses (OR=1.896, 95% CI=1.028, 3.496). There was no statistical significance between the stress and the other study variables, as shown in Table 5.

The likelihood of developing poor sleep quality was higher in nurses who were younger than 36 years of age (OR=1.922, 95% CI=1.208, 3.060), unmarried nurses (OR=2.174, 95% CI=1.294, 3.651), and nurses that have less than 10 years of experience (OR=1.934, 95% CI=1.203, 3.109). No statistical significance between the poor quality of sleep and the other study variables was found, as shown in Table 5.

**Table 5:** Relation between stress, quality of sleep, and study variables.

Variables	P value	Stress OR	95% CI of OR		Quality of sleep	
				p-value	OR	95% CI of OR
Age (years)	0.002	2.675	1.397, 5.125	0.006	1.922	1.208, 3.06
<36						
>36						
Gender	0.497	0.735	0.301, 1.792	0.126	0.540	0.243, 1.201
Female						
Male						
Nationality	0.082			<0.0001		
Filipino		1 (Reference)			1 (Reference)	
Indian		2.095	0.799, 5.493		3.551	2.122, 5.942
others		3.341	1.125, 9.917		1.319	0.529, 3.288
Marital status	0.039	0.528	0.286, 0.973	0.003	0.460	0.274, 0.773
Married						
Not married						
Work experience (years)	0.069	1.746	0.954, 3.196	0.006	1.934	1.203, 3.109
<10						
>10						
Clinical department	0.131			0.063		
Medicine		1 (Reference)			1 (Reference)	
Ambulatory care		0.839	0.331, 2.126		1.559	0.779, 3.119
ICU		0.422	0.182, 0.979		0.926	0.454, 1.887
Women’s health and pediatric		1.581	0.481, 5.194		1.410	0.663, 3.002
Surgery		1.089	0.358, 3.316		0.376	0.148, 0.955
Others		1.335	0.443, 4.023		0.753	0.349, 1.625
Live status	0.586	1.192	0.634, 2.242	0.074	1.582	0.954, 2.623
Alone						
Not alone						
Frequency of contact with COVID-19 patients	0.369	1.319	0.72, 2.418	0.997	0.999	0.625, 1.596
Often/Very often Never/Rarely						
COVID-19 Infection	0.679	1.162	0.570, 2.372	0.652	0.880	0.505, 1.533
Infected						
Not infected						
Working hours	t-value	P value	95% CI of deference mean	t-value	P value	95% CI of deference mean
	-1.047	0.296	-0.682, 0.208	0.101	0.135	-0.081, 0.597

## Discussion

This study demonstrated a high prevalence of mental illnesses and poor quality of sleep symptoms among nurses working at KKUH during the COVID-19 pandemic. This suggests that poor mental health and poor quality of sleep were positively associated with COVID-19 patient care and quarantine/self-isolation experience. According to the present results, 30.4% of the nurses had symptoms of depression. Similar findings were reported in a study conducted in Riyadh, Saudi Arabia, which included 281 nurses, 29.2% of which showed moderate/severe depression [5]. Some studies showed a lower prevalence of depression symptoms [6,8]. Conversely, other studies showed a higher prevalence [7,9,12,19].

Regarding anxiety, 43.7% of nurses had symptoms of anxiety, and the results of the present study are in accordance with a study conducted in China [12], reporting that 40% showed symptoms of anxiety. Other studies, in Saudi Arabia and Iran, revealed a lower percentage of nurses suffering from anxiety [6,9,19]. In contrast, another study showed a higher anxiety percentage of 57% among nurses compared to the percentage reported in the present study [5]. Regarding stress, in our study, we found that 16.5% of nurses had stress symptoms. Balay-Odao et al. reported a percentage of moderate to severe stress (12%), lower than that reported in the present study [5]. In contrast, Arafa et al. reported a higher percentage of stress (53.4%) among nurses [7]. Regarding the quality of sleep, 61.5% of nurses reported poor sleep quality. Likewise, two studies were performed in Saudi Arabia and China, reporting 65.6% and 60% poor quality of sleep, respectively, the results of which are in accordance with those of the present [11,12]. Few studies observed a percentage of poor-quality sleep higher than that reported in this study [4,13]. It should be noted that different tools of evaluation and different classifications can result in very different numbers for the prevalence of mental illnesses [6]. The differences between these results and previous reports could be explained by the different conditions of work [15].

In the present study, we found that nurses younger than 36 years old with less than 10 years of work experience, besides being unmarred are more likely to have mental illnesses and poor quality of sleep. This indicates that married older nurses with more than 10 years of work experience have a lower likelihood of developing mental illness. It may be that older nurses had more experience and were better prepared both professionally and mentally to cope with the stress during the pandemic. The results indicate that nurse managers could increase COVID-19 training and support to younger, less-experienced staff for mental health problems to be reduced [16]. Others have found similar results with a positive correlation between previously mentioned demographic variables and mental illness and poor quality of sleep [6-8,16]. However, there was a negative correlation between mental illness and poor quality of sleep and other variables such as gender, nationality, clinical departments, working hours, and frequency of contact with COVID-19-infected patients.

Anxiety and depression symptoms can be caused by a lack of support from friends and family, stress at the workplace, and concern about COVID-19 [20-22]. Poor sleep can be caused by a heavy workload, negative perceptions such as the fear of infecting others, and a lack of knowledge of COVID-19 [22]. Nurses represent almost 50% of global healthcare workers, and it is possible that the findings of this study will be similar to other healthcare professionals who work and live in similar conditions. Therefore, it is important that, in a potential second wave of the COVID-19 outbreak, improving working conditions can help protect nurses' mental health and enhance their sleep as well [3].

The significant association between poor quality of sleep and the other outcome variables of mental health (depression, anxiety, and stress) showed the importance of evaluating the mental and sleep problems among nurses during pandemics, such as COVID-19. Sleep deprivation negatively affects the nurse's performance and can cause accidents and errors during work [23]. Evidence-based studies revealed that interventions by psychiatrists can help them cope with mental health and sleep disorders during pandemics [24]. As there is an association between poor sleep quality and mental health illnesses, improving sleep quality will lead to reduced mental illness prevalence.

Regarding the limitations of this study, the cross-sectional study type might influence the causal relationship between the sociodemographic and outcome variables (mental health, poor sleep quality). For example, the majority of participants (89%) were female, and the rest (11%) were male. Another limitation was the self-reported measurement tool, which may cause under-/overestimation by the participants, leading to information bias. Thus, the results may have been influenced by self-selection bias, as nurses who had higher levels of depression or anxiety and poor sleep quality were more likely than others to respond.

Furthermore, the present study used DASS-21 and PSQI to measure the nurses' sleep, which might not be very objective. Moreover, because of the snowball sampling technique used in this study, it may be impossible to determine an exact response. Finally, the study was not conducted at the peak period of the pandemic in Saudi Arabia, and it was conducted in one hospital; thus, the generalization is limited. In addition to the factors studied, there could be other factors affecting mental health and sleep quality.

## Conclusions

In conclusion, nurses have reported an increased prevalence of depression, anxiety, stress, and poor quality of sleep during the COVID-19 pandemic, making them particularly vulnerable to mental illnesses and sleep difficulties in the event of future pandemics. There is a clear link between mental health issues and poor quality of sleep, which necessitates the provision of psychological support for nurses. Enhancing sleep quality is recommended to decrease the prevalence of mental health concerns.

Future studies are recommended to assess the causal relationship between outcome variables by using different study designs. Furthermore, the long-term mental health impact of the COVID-19 pandemic among nurses needs to be addressed. To ensure the generalization factor, future studies should be performed in more than one hospital, in different regions of Saudi Arabia.
